# Temporal Trends in Mortality Related to Enteritis and Septicaemia in the United States

**DOI:** 10.7759/cureus.94513

**Published:** 2025-10-13

**Authors:** Aditya Bikram Raychaudhuri, Sonali Priya Sahu, John Jose Tania, Nanthan Paramaguru Sugumaran, Ferlin Selvaraj

**Affiliations:** 1 General Surgery, Manipal University College, Melaka, MYS; 2 Surgery, The Oxford Medical College, Hospital and Research Centre, Bangalore, IND; 3 General Surgery, Chettinad Hospital and Research Institute, Kelambakkam, IND; 4 General Medicine, Tiruppur Government Medical College Hospital, Tiruppur, IND; 5 General Surgery, University of Visayas Gullas College of Medicine, Cebu, PHL

**Keywords:** age-adjusted mortality rate, cdc wonder mcd database, non-infective enteritis and colitis, retrospective study, septicaemia

## Abstract

Introduction: Non-infective enteritis and colitis with septicemia as a contributing cause remains an underexplored area of mortality research. While previous studies have examined these conditions independently, understanding their concurrent occurrence and combined impact on mortality is essential to identify high-risk groups and inform prevention strategies.

Aims: To analyze temporal mortality trends and demographic disparities in non-infective enteritis and colitis with septicemia as a contributing cause in the United States using the CDC Multiple Cause of Death (MCD) database from 1999 to 2020.

Methodology: A retrospective observational study was conducted using the CDC MCD database for individuals aged ≥25 years. Deaths were identified where non-infective enteritis and colitis (International Classification of Diseases, Tenth Revision (ICD-10): K50-K52) were listed as the underlying cause and septicemia (ICD-10: A41) as a contributing cause. Trends were analyzed by sex, race, metropolitan status, and place of death using Joinpoint regression.

Results: A total of 12,143 deaths were recorded, corresponding to a crude mortality rate of 2.7 per 1,000,000 population. The age-adjusted mortality rate (AAMR) increased significantly between 1999-2007 (Annual Percent Change [APC]: +7.13%), declined sharply between 2007-2010 (APC: −39.52%), and gradually decreased thereafter (APC: −2.96%). Mortality was highest among females (61.9%), White individuals (89.8%), residents of metropolitan areas (84.4%), and deaths occurring in medical facilities (88.8%).

Conclusions: Mortality related to non-infective enteritis and colitis with septicemia showed an initial rise followed by a marked decline and later stabilization over two decades. Disparities were noted across sex, race, and geographic location, underscoring the need for improved early recognition and management of septicemia in high-risk enteritis and colitis patients.

## Introduction

Enteritis refers to the inflammation of the small intestine. Each year, there are approximately 350 million cases of acute gastroenteritis globally, with about 48 million attributed to foodborne bacteria in the United States [1]. Risk factors include recent household stomach flu, travel, and exposure to contaminated water [2]. Symptoms can begin within hours to days after infection and may include abdominal pain, severe diarrhea, loss of appetite, vomiting, and blood in the stool, with potential complications such as dehydration and prolonged diarrhea [2,3].

In severe instances of enteritis caused by Salmonella enteritidis, the infection may progress to septicemia, which can result in rapid clinical deterioration due to hemorrhagic necrotic enteritis, acute kidney failure, and sepsis [4]. Septicemia poses a significantly greater risk of death compared to enteritis alone, often with mortality rates several times higher. While enteritis is typically self-limiting, once it advances to a bloodstream infection, the mortality rate can increase dramatically, reaching 25% or more.

Although enteritis-related deaths were historically low, they increased during the late 20th century, particularly due to diarrheal diseases affecting older adults and infants. Meanwhile, sepsis-related mortality rose sharply during the late 1970s and 1980s, with incidence continuing to climb throughout the 2000s [5,6].

Despite advances in medicine, enteritis and septicemia continue to cause a significant number of deaths worldwide and nationally, particularly among vulnerable populations such as infants, the elderly, and immunocompromised individuals. This study aims to understand the mortality burden associated with enteritis and septicemia in the United States to guide healthcare policy and improve preventive strategies.

The aim of this study is to analyze temporal changes in mortality rates from 1999 to 2020 among adults aged 25 years and older in the United States, focusing on deaths where non-infective enteritis and colitis (International Classification of Diseases, Tenth Revision (ICD-10): K50-K52) were recorded as the underlying cause and septicaemia (ICD-10: A41) as a contributing cause. The objective is to evaluate trends in age-adjusted mortality, examine demographic and geographic variations by age, sex, race, and ethnicity, and interpret their public health significance in improving early recognition and management of septicemia in patients with non-infective enteritis and colitis.

## Materials and methods

This retrospective, population-based observational study analyzed mortality data obtained from the Centers for Disease Control and Prevention (CDC) Wide-ranging Online Data for Epidemiologic Research (WONDER) Multiple Cause of Death (MCD) database. The database compiles death certificate information from all states and territories of the United States. The study period extended from January 1, 1999, to December 31, 2020. The study was conducted in accordance with the Strengthening the Reporting of Observational Studies in Epidemiology (STROBE) guidelines for cross-sectional and time-trend studies. Ethical approval was not required as the dataset is publicly available, de-identified, and does not involve direct human participation.

The study population comprised all deaths registered in the United States within the defined period. The analysis was restricted to adults aged 25 years and older. Death records were included if non-infective enteritis and colitis, coded as K50-K52 under the ICD-10, were listed as the underlying cause of death and if septicaemia, coded as A41, was recorded as a contributing cause of death. Records with incomplete demographic or geographic information, missing ICD-10 codes, or those pertaining to individuals below 25 years of age were excluded from the analysis.

The primary outcome variable was mortality associated with non-infective enteritis and colitis as the underlying cause of death with septicaemia as a contributing cause. The variables analyzed included age, sex, race, ethnicity, metropolitan or non-metropolitan residence, and place of death, such as medical facility, nursing home, residence, hospice, or other categories. Diagnostic classification was based on ICD-10 codes as per CDC WONDER standards. The year of death, from 1999 to 2020, was used as the temporal variable to assess changes in mortality trends over time.

Data were extracted from the CDC WONDER MCD database using the “Underlying and Multiple Cause of Death” function, and finalized data within the study period were included. The extracted dataset was downloaded in comma-separated values (CSV) format for further analysis. Data cleaning and organization were performed using Microsoft Excel (Microsoft Corp., Redmond, WA, USA) and R software (R Foundation for Statistical Computing, Vienna, Austria).

Potential sources of bias, including misclassification or underreporting of causes of death, were minimized by using nationally standardized CDC data. Since all U.S. jurisdictions adhere to uniform coding practices, classification bias was limited. However, potential inaccuracies inherent to death certificate reporting were recognized and addressed in the discussion as study limitations.

Crude and age-adjusted mortality rates were calculated using the 2000 U.S. Standard Population for age standardization. Temporal trends were analyzed using Joinpoint Regression Software (Version 5.0.2; National Cancer Institute, Bethesda, MD, USA) to identify statistically significant inflection points in mortality rates. Annual Percent Change (APC) and corresponding 95% confidence intervals (CIs) were computed to determine the direction and magnitude of mortality trends. Statistical analyses were performed separately for the overall population and subgroups by sex and race. Descriptive analysis and data visualization were carried out using Microsoft Excel and R software. Statistical significance was determined at a p-value of less than 0.05.

Because the study utilized publicly available, anonymized data, institutional review board (IRB) approval was not required. The study was conducted in accordance with the principles of transparent reporting and reproducibility, as recommended by the STROBE Statement.

## Results

According to the CDC MCD database, a total of 12,143 deaths occurred among individuals aged 25 years and older in the United States between 1999 and 2020, where non-infective enteritis and colitis were identified as the underlying cause and septicaemia as a contributing cause. The crude mortality rate was 2.7 per 1,000,000 population.

Demographic characteristics

Of the total deaths, 7,518 (61.9%) occurred in females and 4,625 (38.1%) in males. Mortality was highest among White individuals (n = 10,906; 89.8%), followed by Black or African American individuals (n = 1,009; 8.3%), Asian or Pacific Islander individuals (n = 167; 1.4%), and American Indian or Alaska Native individuals (n = 61; 0.5%).

Geographic and place-of-death characteristics

Most deaths occurred in metropolitan areas (n = 10,248; 84.4%), compared with non-metropolitan areas (n = 1,895; 15.6%). The majority of deaths occurred in medical facilities (n = 10,771; 88.8%), followed by nursing homes or long-term care facilities (n = 636; 5.2%), residences (n = 354; 2.9%), and hospice facilities (n = 231; 1.9%).

Temporal trends

**Figure 1:**
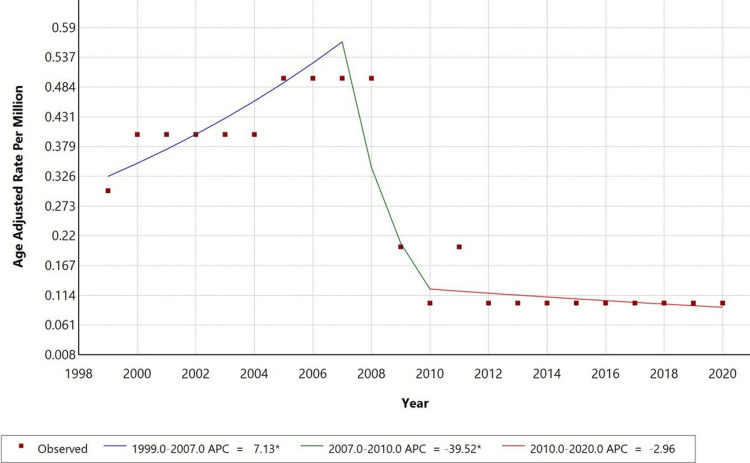
Overall age-adjusted mortality rates among adults aged 25+ in the United States, 1999 to 2020. *Indicates that the Anuual Percentage Change (APC) is significantly different from zero at alpha=0.05 level.

The age-adjusted mortality rate (AAMR) for non-infective enteritis and colitis with septicaemia as a contributing cause exhibited three distinct phases during 1999-2020 (Figure 1). From 1999 to 2007, AAMR increased significantly (APC: +7.13%, p < 0.05), followed by a sharp decline from 2007 to 2010 (APC: −39.52%, p < 0.05), and a gradual decrease thereafter (2010-2020) (APC: −2.96%, p < 0.05). 

**Figure 2:**
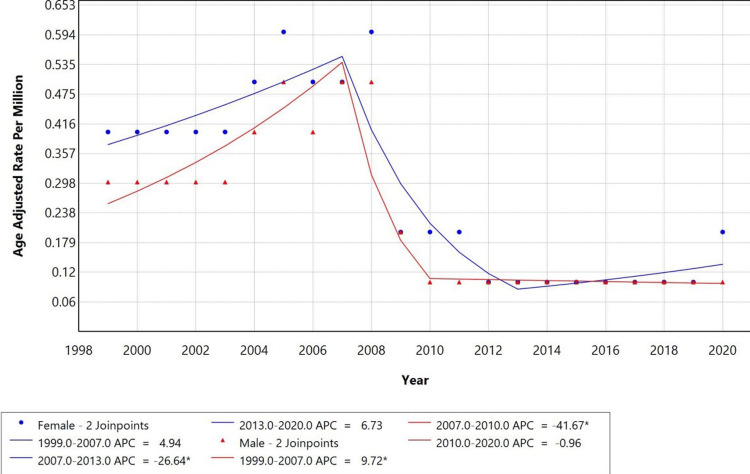
Trends in sex-stratified age-adjusted mortality rates among adults aged 25+ in the United States, 1999 to 2020. *Indicates that the Annual Percentage Change (APC) is significantly different from zero at alpha=0.05 level.

When stratified by sex (Figure 2), mortality among females increased from 1999-2007 (APC: +4.94%), declined significantly between 2007-2013 (APC: −26.64%), and showed a moderate increase thereafter (APC: +6.73%). Among males, mortality rose sharply from 1999-2007 (APC: +9.72%), declined between 2007-2010 (APC: −41.67%), and remained stable thereafter (APC: −0.96%).

**Figure 3:**
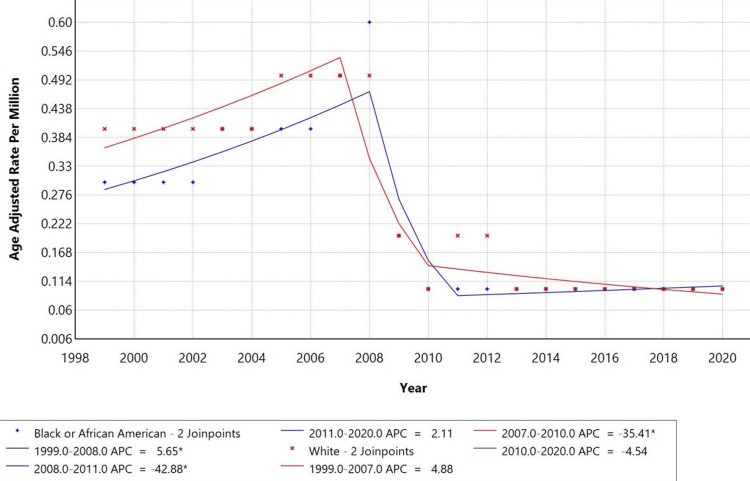
Trends in age-adjusted mortality rates stratified by race among adults aged 25+ years in the United States, 1999 to 2020. *Indicates that the Anuual Percentage Change (APC) is significantly different from zero at alpha=0.05 level. Temporal trends for American Indian/Alaska Native and Asian or Pacific Islander are not displayed due to data suppression for counts <10, limiting reliable trend analysis.

Racially stratified trends (Figure 3) revealed differing patterns. Among Black or African American individuals, mortality increased from 1999-2008 (APC: +5.65%), declined sharply between 2008-2011 (APC: −42.88%), and then rose slightly through 2020 (APC: +2.11%). Among White individuals, mortality rose from 1999-2007 (APC: +4.88%), declined significantly between 2007-2010 (APC: −35.41%), and decreased gradually thereafter (APC: −4.54%). Trends for American Indian/Alaska Native and Asian or Pacific Islander groups were not plotted due to data suppression for counts fewer than 10, precluding reliable analysis.

## Discussion

This retrospective study utilized the CDC MCD database to evaluate national mortality trends for noninfective enteritis and colitis (ICD-10: K50-K52) with septicemia (ICD-10: A41) as a contributing cause of death in the United States from 1999 to 2020, focusing on adults aged 25 years and older. Across this 22-year period, a total of 12,143 deaths were recorded with noninfective enteritis and colitis as the underlying cause. The majority of these deaths occurred in medical facilities (88.8%) and metropolitan areas (84.4%), with the highest mortality seen in females (61.9%) and white individuals (89.8%). AAMRs showed statistically significant temporal changes over time, with differing patterns observed by sex and race. Mortality was concentrated in large central metropolitan areas, suggesting a potential intersection of disease burden and healthcare access.

Noninfective enteritis and colitis, including Crohn’s disease and ulcerative colitis, compromise intestinal mucosal integrity, predisposing individuals to systemic infections such as septicemia. The loss of mucosal barrier function during disease flares or complications such as perforation or toxic megacolon can facilitate bacterial translocation into the bloodstream [7]. In addition, immunosuppressive therapies commonly used in inflammatory bowel disease (IBD), such as corticosteroids, thiopurines, and biologics, further increase the risk of opportunistic infections [8,9]. Septicemia contributes significantly to mortality in these cases through overwhelming systemic inflammation, multiorgan dysfunction, and rapid clinical deterioration [10]. The relationship between IBD and infection-related mortality has been well-documented in hospitalized cohorts and is especially prominent among older adults and those undergoing surgery [11].

The overall AAMR for noninfective enteritis and colitis with septicemia in this study was 2.7 per million, showing distinct temporal shifts across the study period. These findings are consistent with prior studies showing increased rates of sepsis-related deaths among IBD patients, particularly in older adults and those treated with immunomodulatory agents [12,13]. Colbert JF et al. reported higher in-hospital mortality in IBD patients with sepsis compared to those without [11], while others have highlighted differences in outcomes between ulcerative colitis and Crohn’s disease. Some earlier studies have shown declining mortality trends in IBD due to improvements in treatment and infection control [14], though these gains may be offset by newer risks such as antimicrobial resistance and polypharmacy [13].

Gender differences observed in this study, with females accounting for a higher proportion of deaths, may reflect demographic distributions, health-seeking behaviors, or reporting patterns, rather than true biological susceptibility. Further investigation is needed to understand these variations in larger, clinically detailed cohorts [15]. Racial disparities were also evident. Although White individuals made up the majority of deaths, Black or African American individuals experienced disproportionately high age-adjusted mortality rates relative to their population size. Prior studies have linked racial disparities in IBD outcomes to factors such as delayed diagnosis, underrepresentation in clinical trials, and reduced access to specialty care [16,17].

Geographic disparities were also observed, with most deaths recorded in metropolitan areas (84.4%). This likely reflects better reporting systems, higher concentration of healthcare facilities, and diagnostic accessibility in urban centers, rather than a true increase in disease prevalence. In contrast, non-metropolitan areas may experience underdiagnosis and limited access to specialized care [9,17].

Temporal trend analysis revealed statistically significant changes in AAMRs from 1999 to 2020, with distinct patterns by gender and race. These dynamic trends mirror national shifts in healthcare utilization, diagnostic coding practices, and treatment access. Advances in IBD therapy, particularly the introduction of biologics and targeted immunosuppressants, may have initially contributed to reduced mortality, but these same therapies carry infectious risks that can elevate septicemia-related deaths [7,13]. Racial differences in trends, likely influenced by social determinants of health and healthcare disparities, warrant closer investigation in future work.

These findings have important public health implications. There is a need for early identification of sepsis in patients with noninfective enteritis and colitis, particularly among high-risk groups such as the elderly and immunosuppressed. Future studies should explore disparities in treatment access, investigate the effectiveness of sepsis prevention strategies in IBD populations, and evaluate regional care delivery models. Policy interventions targeting healthcare equity, antimicrobial stewardship, and access to specialty care, especially in rural or underserved populations, will be essential in addressing preventable mortality in this high-risk group.

Limitations

This study has several limitations. First, death certificate data may contain coding errors and misclassification bias. Second, the MCD database lacks clinical details such as comorbidities, treatment history, and disease severity. Third, temporal changes in diagnostic and reporting practices may have influenced observed trends. Fourth, the retrospective and observational design precludes causal inference. Lastly, important confounders such as socioeconomic status, healthcare access, and lifestyle factors were not available in the dataset and may have affected mortality outcomes.

## Conclusions

This study demonstrates that mortality due to non-infective enteritis and colitis with septicemia as a contributing cause in the United States showed a distinct temporal pattern from 1999 to 2020 - an initial rise, followed by a sharp decline, and subsequent stabilization. Mortality was higher among females and White individuals, with most deaths occurring in metropolitan areas and medical facilities.

These findings highlight the importance of understanding coexisting gastrointestinal and infectious conditions through continuous surveillance and data-driven approaches. Targeted public health strategies focusing on early detection, infection control, and equitable access to care are needed to reduce mortality in high-risk populations. Further research should explore underlying biological, demographic, and healthcare-related factors contributing to these observed disparities.
